# Hybrid Materials Based on Carbon Nanotubes and Tetra- and Octa-Halogen-Substituted Zinc Phthalocyanines: Sensor Response Toward Ammonia from the Quantum-Chemical Point of View

**DOI:** 10.3390/s25010149

**Published:** 2024-12-30

**Authors:** Pavel Krasnov, Victoria Ivanova, Darya Klyamer, Dmitry Bonegardt, Aleksandr Fedorov, Tamara Basova

**Affiliations:** 1 International Research Center of Spectroscopy and Quantum Chemistry, Siberian Federal University, 26 Kirensky St., 660074 Krasnoyarsk, Russia; kpo1980@gmail.com; 2 Qingdao Innovation and Development Center, Harbin Engineering University, 1777 Sansha St., Huangdao Dist., Qingdao 266500, China; 3 Nikolaev Institute of Inorganic Chemistry SB RAS, 3 Lavrentiev Pr., 630090 Novosibirsk, Russia; vivanova@niic.nsc.ru (V.I.); klyamer@niic.nsc.ru (D.K.); bonegardt@niic.nsc.ru (D.B.); 4 Kirensky Institute of Physics, Federal Research Center KSC SB RAS, 50/38 Akademgorodok, 660036 Krasnoyarsk, Russia; qchem99@yandex.ru

**Keywords:** zinc phthalocyanines, carbon nanotubes, quantum-chemical calculations, DFTB, band structure, electrical conductivity, ammonia sensors

## Abstract

This paper presents the results of quantum-chemical modeling performed by the Density Functional-Based Tight Binding (DFTB) method to investigate the change in the band structure of hybrid materials based on carbon nanotubes and unsubstituted, tetra-, or octa-halogen-substituted zinc phthalocyanines upon the adsorption of ammonia molecules. The study showed that the electrical conductivity of these materials and its changes in the case of interaction with ammonia molecules depend on the position of the impurity band formed by the orbitals of macrocycle atoms relative to the forbidden energy gap of the hybrids. The sensor response of the hybrids containing halogenated phthalocyanines was lower by one or two orders of magnitude, depending on the number of substituents, compared to the hybrid with unsubstituted zinc phthalocyanine. This result was obtained by calculations performed using the nonequilibrium Green’s functions (NEGF) method, which demonstrated a change in the electrical conductivity of the hybrids upon the adsorption of ammonia molecules. The analysis showed that in order to improve the sensor characteristics of CNT-based hybrid materials, preference should be given to those phthalocyanines in which substituents contribute to an increase in HOMO energy relative to the unsubstituted macrocycles.

## 1. Introduction

Carbon nanotubes (CNTs) have been receiving a lot of attention due to their unique physical properties. These properties can be enhanced by creating hybrid nanostructures that combine two or more different nanostructured elements. These hybrid materials based on CNTs have improved mechanical, thermal, optical, and electrical properties compared to single-component materials [1,2,3]. There is a significant body of literature on the use of CNTs and their hybrids in various applications. For example, they have been proposed as potential candidates for use in photovoltaic devices [4,5,6,7], energy storage systems [8,9], fuel cells [10,11,12], and environmental applications [13,14,15]. Another important application of carbon nanotubes is in chemical sensors, mainly electrochemical and chemiresistive ones [16,17,18]. CNTs and their hybrids are widely used as active layers of chemiresistive sensors for the detection of various gases, e.g., NO_x_, H_2_S, Cl_2_, and NH_3_, in the environment or gas mixtures [19,20,21]. Much research in this area focuses on the detection of gaseous ammonia, the control of which plays an important role in the environmental monitoring of air and soils [22,23,24].

Metal nanoparticles [25,26,27], TiO_2_ nanomaterials [22], polymers [28,29,30], and polyaromatic molecules [31,32,33] are widely used to modify carbon nanotubes. Among the variety of hybrid materials with conjugated aromatic molecules, CNTs modified with metal phthalocyanines (MPc) have received significant attention. Since the non-covalent functionalization of CNTs with MPc molecules does not violate the electronic structure of nanotubes and is an easy way to obtain hybrid materials, most hybrid materials based on carbon nanotubes are obtained by adsorption of phthalocyanines on the surface of CNTs due to π-π-interaction [34]. The literature contains numerous examples of studies of CNT/MPc hybrids and their layers as active layers of electrocatalytic [35,36], optoelectronic [37,38], and photovoltaic devices [39,40], and chemical sensors [41,42,43]. Modification of carbon nanotubes with cobalt phthalocyanine (CoPc) significantly improves not only their catalytic activity and current density but also the selectivity and stability of the devices [44]. Moreover, it has been shown that the thermoelectric properties of a hybrid based on CNT and copper phthalocyanine (CuPc) are superior to those of either component alone [45]. In addition, CNT/MPc hybrids are also widely used as active layers of chemiresistive gas sensors [19,20,21], including sensors for ammonia detection [43,46].

The sensor properties of CNT/MPc hybrids toward ammonia can be improved by modifying metal phthalocyanine by introducing substituents to their peripheral positions instead of hydrogen atoms. In particular, we have previously shown that the introduction and increase in the number of pyrene substituents into MPc led to the increase in the sensor response of CNT/MPc hybrids to ammonia [47,48]. This was due to a change in the band structure of hybrids, in which the valence band top and conduction band bottom were formed by the orbitals of carbon nanotube atoms. The orbitals, in particular, of zinc phthalocyanine atoms form an impurity level located in the hybrid forbidden band. The difference in energy between this level and the conduction band bottom determines the thermal population of the conduction band with electrons and, as a result, electrical conductivity. When ammonia molecules adsorb, this difference increases (electrical conductivity decreases), and its change is more significant the more pyrene substituents in zinc phthalocyanine (ZnPc) are on the carbon nanotube surface [48].

It was recently shown that substituting hydrogen atoms with electron-accepting halogens, such as fluorine or chlorine, can increase the sensor response to electron-donating gases like ammonia [49,50,51,52,53,54]. Fluorine and chlorine substituents reduce the electron density of the aromatic ring and increase the oxidative potential of the MPc molecule, thereby making it more sensitive to reducing gases [55]. For example, it was found that the chemiresistive response of the MPcF_4_ (M = Zn, Co, Cu) films to NH_3_ was 30–70 times higher than that of the corresponding MPc films [51]. ZnPcCl_4_ films turned out to be even more sensitive to ammonia than MPcF_4_ [53]. They also had an incredibly low detection limit, which could reach 0.01 ppm. At the same time, hybrid materials based on carbon nanotubes and halogenated phthalocyanines have not been studied as sensors for ammonia. In this regard, the study of the effect of this halogen substitution on the sensor response of MPc hybrid materials with carbon nanotubes to ammonia seems relevant.

This paper presents the results of quantum-chemical calculations of the change in the band structure and electrical conductivity of hybrid materials of carbon nanotube CNT(10,0) with zinc phthalocyanines bearing 4 and 8 fluorine or chlorine substituents in peripheral positions of their macrocycle when interacting with ammonia molecules. Studies have shown that contrary to expectations, halogenated zinc phthalocyanines led to a decrease in the sensor response of their hybrids to ammonia compared to those with unsubstituted phthalocyanines. These results may help determine the optimal structure of phthalocyanines, which, in turn, will improve the sensor properties of carbon nanotube hybrid materials.

## 2. Objects and Methods of Investigation

All quantum-chemical calculations in the work were performed by the Density Functional Tight Binding (DFTB) method in the approximation of the self-consistent charge (SCC-DFTB), using the DFTB+ software package (release 24.1) [56,57], 3OB interatomic interaction parameters (Slater–Koster files) [58,59], and DFT-D3 dispersion interaction correction [60,61]. The electrical conductivity of the considered hybrid materials was estimated based on calculations of the dependence of the transmission coefficient on the electron energy, performed within the framework of the nonequilibrium Green’s function (NEGF) method [62].

**Figure 1:**
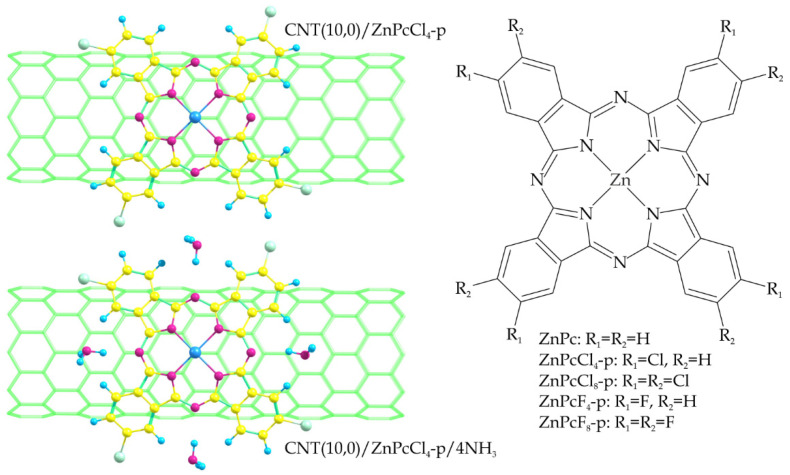
Examples of a geometric structure of optimized compounds under consideration—CNT(10,0)/ZnPcCl_4_-p (**top left**) and CNT(10,0)/ZnPcCl_4_-p/NH_3_ (**bottom left**). Structural formula of zinc phthalocyanine derivatives considered in the work (**right**).

In this work, five derivatives that differ in the nature and number of substituents in the peripheral positions were considered, namely, unsubstituted zinc phthalocyanine (ZnPc), tetra-chloro- and tetra-fluoro-substituted molecules (ZnPcCl_4_-p and ZnPcF_4_-p), and octa-chloro- and octa-fluoro-substituted molecules (ZnPcCl_8_-p and ZnPcF_8_-p) (Figure 1). These macrocyclic molecules were located on the surface of a CNT(10,0) carbon nanotube modeled as a supercell consisting of 6 unit cells. This nanotube was chosen because it is a semiconductor, and its hybrids with phthalocyanines have been considered in previous works [47,48,63], allowing us to compare some of our results presented here with those obtained earlier.

Quantum-chemical calculations were performed considering the periodicity of CNT along its axis. A vacuum gap of 60 Å was specified along the other two perpendicular directions to exclude interactions between objects from neighboring cells. Geometry optimization of all considered compounds was carried out until the value of forces acting on atoms reached 1 × 10^−4^ a.u. The k-point samplings of the first Brillouin zone were chosen according to the Monkhorst-Pack scheme [64] as a 1 × 1 × 15 mesh (the CNT axis is directed along the *z*-axis) in the case of pristine CNT(10,0) and its hybrids with phthalocyanines and ammonia molecules and a 1 × 1 × 1 mesh (Gamma-point only) in the case of individual molecules of phthalocyanine and ammonia.

In the first stage, the geometry of the CNT(10,0) supercell was optimized, including the position of the atoms and supercell length, the value of which ultimately amounted to 25.703 Å. Then, its hybrids with zinc phthalocyanine derivatives on the surface (CNT(10,0)/ZnPc, CNT(10,0)/ZnPcCl_4_-p, etc.) were modeled. The orientation of the macrocycles was selected to maximize the overlap of their *π*-orbitals with the *π*-system of the nanotube (Figure 1), based on previous results [65,66]. The geometry of hybrids (relaxation of the positions of the atoms) was optimized at a fixed value of the length of the CNT(10,0) supercell. The obtained results were then used to analyze the band structure and estimate the binding energy *E_b_*(MC) of each macrocycle (MC) with a carbon nanotube as a difference in the total energies of the entire hybrid and its two components:

*E_b_*(MC) = *E*(CNT(10,0)/MC) − *E*(CNT(10,0)) − *E*(MC). (1)

In the second stage, the geometry of the hybrids under consideration was optimized with four adsorbed ammonia molecules (CNT(10,0)/ZnPc/4NH_3_, CNT(10,0)/ZnPcCl_4_-p/4NH_3_, etc.), which were bound to the macrocycles by forming hydrogen bonds with the bridging nitrogen atoms and hydrogen atoms in non-peripheral positions of the macrocycle (Figure 1). This method of binding NH_3_ molecules to a phthalocyanine is considered the most probable for describing the mechanism of the sensor response of such hybrids [47,48,53,54]. This type of interaction of NH_3_ molecules with phthalocyanines was also confirmed by experimental studies carried out by Chia et al. [67]. The authors used in situ X-ray absorption spectroscopy (XAS) to investigate the interaction of unsubstituted copper phthalocyanine CuPc with NH_3_. The observations did not show that NH_3_ was coordinated with the copper center. However, based on the data investigating the extended X-ray absorption fine structure (EXAFS), it has been suggested that ammonia interacted with atoms through a benzene ring or a nitrogen bridge atom.

In our case, the interaction energy is quite low, typically a few tenths of eV for hydrogen and such elements as nitrogen and oxygen, which is less than the energy required for covalent bonding (on the order of units of eV). This ensures easy desorption of ammonia, resulting in a reversible sensor response. On the other hand, it is higher than the energy of dispersion interaction (several hundredths or even thousandths of eV, typically below 0.03 eV), which means that the change in the band structure and measurable sensitivity of the hybrids, which are negligible in the case of physical sorption, should be observed. Thus, earlier we considered the interaction of ammonia molecules with hybrids via a zinc atom [66]. The absolute value of the energy for this interaction was about 0.8 eV, which corresponded to the formation of coordination bonds [68,69] and indicated that the sensor response should be irreversible. However, experimental studies showed that the response was reversible [47].

Geometry optimization of the CNT(10,0)/MC/4NH_3_ compounds was also performed to relax the position of all atoms at a fixed value of the CNT(10,0) supercell length. Then, the band structure of these compounds was analyzed, and using the obtained values of the total energy of the corresponding hybrid with four ammonia molecules, without NH_3_ molecules and only ammonia molecule, the average value of the binding energy *E_b_*(NH_3_) of the NH_3_ molecule was calculated using Equation (2).

(2) $$E_{b}({\mathrm{NH}}_{3})=\frac{1}{4}[E(\mathrm{CNT}(10,0)/\mathrm{MC}4{\mathrm{NH}}_{3})\;-\;E(\mathrm{CNT}(10,0)/\mathrm{MC})\;-\;4E({\mathrm{NH}}_{3})].$$

In the third stage, the NEGF method was used to calculate the dependence of the electrons’ transmission coefficient on their energy in the CNT(10,0)/MC and CNT(10,0)/MC/4NH_3_ aggregates. The obtained values were used to calculate the integral electrical conductivity of these aggregates and conductance change due to the adsorption of ammonia molecules by hybrids. The technical details of these calculations are given below in the relevant section.

## 3. Results and Discussion

As a result of quantum-chemical calculations, it was found that the binding energy of ZnPc with CNT(10,0) is −1.874 eV (Table 1). This value is in good agreement with the results of previous studies, which showed that it can range from −1.610 eV [58] to −2.065 eV [47], depending on the chosen calculation method and the carbon nanotube length. For instance, in [63], CNT(10,0) was considered as a short fullerene-like model closed with caps at both ends, whereas in our work [47], the CNT(10,0) supercell was twice as long as the one considered here and consisted of 12 unit cells.

The introduction of halogen atoms into the peripheral positions of zinc phthalocyanine enhances the interaction between the macrocycle and CNT(10,0) (Table 1). In this case, as the number of substituents increases, the absolute value of *E_b_*(MC) becomes higher, reaching the highest value in the case of chlorine atoms compared to fluorine atoms. This indicates that halogenation of phthalocyanines can be an effective way to increase the degree of functionalization of carbon nanotubes, which, in turn, can improve their sensor properties [33,70].

We showed earlier, using zinc phthalocyanine derivatives with pyrene substituents, that the boundaries of the forbidden energy gap ∆*E*_1_ of their hybrids are formed by the orbitals of carbon nanotube atoms [48]. The orbitals of macrocycle atoms form an impurity level in the forbidden band. The energy difference ∆*E*_2_ between this level and the conduction band bottom determines the energy of the thermal transition of electrons and, as a consequence, the electrical conductivity of the hybrid—the smaller ∆*E*_2_, the higher the conductivity since the concentration of charge carriers in the conduction band is higher. During the adsorption of ammonia, the value of ∆*E*_2_ increases, which leads to a decrease in conductivity. In this case, the change in ∆*E*_2_ is more significant, the more pyrene substituents zinc phthalocyanine has. This fact is consistent with experimental observations, which have shown that hybrids containing phthalocyanines with a larger number of substituents exhibited the highest sensor response to ammonia [47].

**Figure 2:**
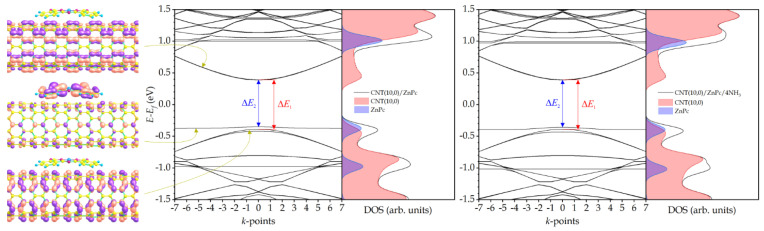
Electronic wave functions of the conduction band bottom, impurity band, and valence band top of the CNT(10,0)/ZnPc hybrid (**left panel**); band structure and DOS of the hybrid without NH_3_ molecules (**center panel**) and with NH_3_ molecules (**right panel**).

The results presented here for CNT(10,0)/ZnPc are consistent with these statements since the calculations were performed using an identical method even though the carbon nanotube supercell is two times shorter. The analysis of the partial densities of states (DOS) demonstrates that the boundaries of the hybrid band gap are formed by the orbitals of the CNT(10,0) atoms, and there is an impurity level in the band gap formed mainly by the orbitals of the macrocycle so that ∆*E*_1_ > ∆*E*_2_ (Figure 2). When four ammonia molecules are added, ∆*E*_1_ does not change, and ∆*E*_2_ increases by 0.009 eV (Table 1). As mentioned above, this should lead to a decrease in the electrical conductivity of the hybrid, which, in turn, determines its sensor response to ammonia.

**Figure 3:**
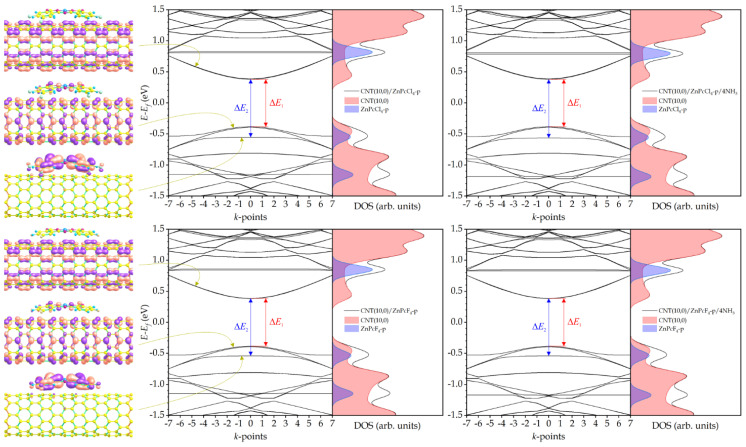
Electronic wave functions of the conduction band bottom, impurity band, and valence band top of CNT(10,0)/ZnPcCl_4_-p and CNT(10,0)/ZnPcF_4_-p hybrids (**left panels**); band structure and DOS of hybrids without NH_3_ molecules (**center panels**) and with NH_3_ molecules (**right panels**).

Unlike the pyrene substituents [48], the introduction of four halogen atoms results in a different shift in the impurity level, which enters the valence band of the hybrid, dropping below its top so that ∆*E*_2_ becomes greater than ∆*E*_1_ (Figure 3). This significantly reduces the possibility of thermal transition of electrons from this level to the conduction band. The adsorption of four ammonia molecules by CNT(10,0)/ZnPcCl_4_-p and CNT(10,0)/ZnPcF_4_-p hybrids has virtually no effect on their band gap (Table 1), although ∆*E*_2_ in the case of CNT(10,0)/ZnPcCl_4_-p increases by 0.010 eV. However, given that the impurity level now plays a much smaller role in the hybrid’s electrical conductivity, this increase is unlikely to significantly alter the ability of CNT(10,0)/ZnPcCl_4_-p to conduct electrical current. As a result, it can be expected that the sensor response of hybrids with tetra-fluoro- and tetra-chloro-substituted zinc phthalocyanines to ammonia will be lower than with unsubstituted ZnPc.

**Figure 4:**
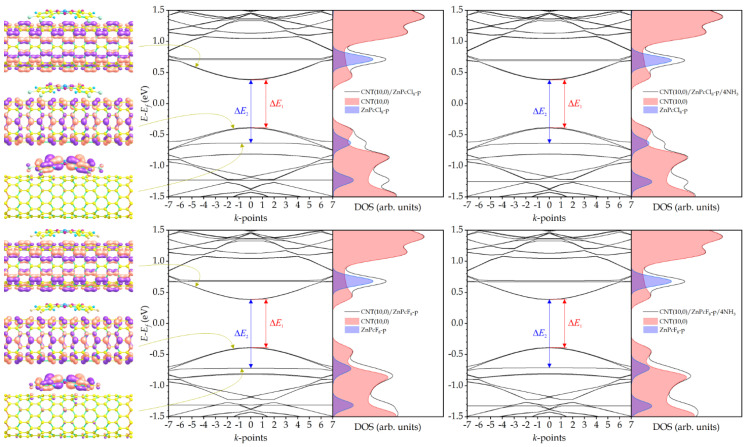
Electronic wave functions of the conduction band bottom, impurity band, and valence band top of CNT(10,0)/ZnPcCl_8_-p and CNT(10,0)/ZnPcF_8_-p hybrids (**left panels**); band structure and DOS of hybrids without NH_3_ molecules (**center panels**) and with NH_3_ molecules (**right panels**).

The introduction of eight halogen atoms into the peripheral positions of ZnPc leads to an even greater shift in the impurity level into the valence band (Figure 4) and, as a consequence, to an even smaller influence of this level on the conductive properties of the hybrids. From this point of view, even though some change in ∆*E*_2_ occurs during the adsorption of ammonia by the CNT(10,0)/ZnPcCl_8_-p and CNT(10,0)/ZnPcF_8_-p compounds, their sensor response should be even lower than in the case of hybrids with tetra-halogen-substituted zinc phthalocyanines.

The above changes in the ∆*E*_2_ value are very small and amount to thousandths of eV. Therefore, it is not clear how significant they are in reality for changing the electrical conductivity of the hybrids. This question is especially relevant for hybrids with halogen-substituted zinc phthalocyanines. In these cases, the influence of the electrons of the impurity level on the overall conductivity should be significantly lower compared to the unsubstituted macrocycles since this level drops in energy below the valence band top. Moreover, in the case of CNT(10,0)/ZnPcF_4_-p and CNT(10,0)/ZnPcF_8_-p, the ∆*E*_2_ value either does not change or decreases when ammonia is added to the hybrids. In this regard, the question arises about the direction of the change in electrical conductivity.

**Figure 5:**
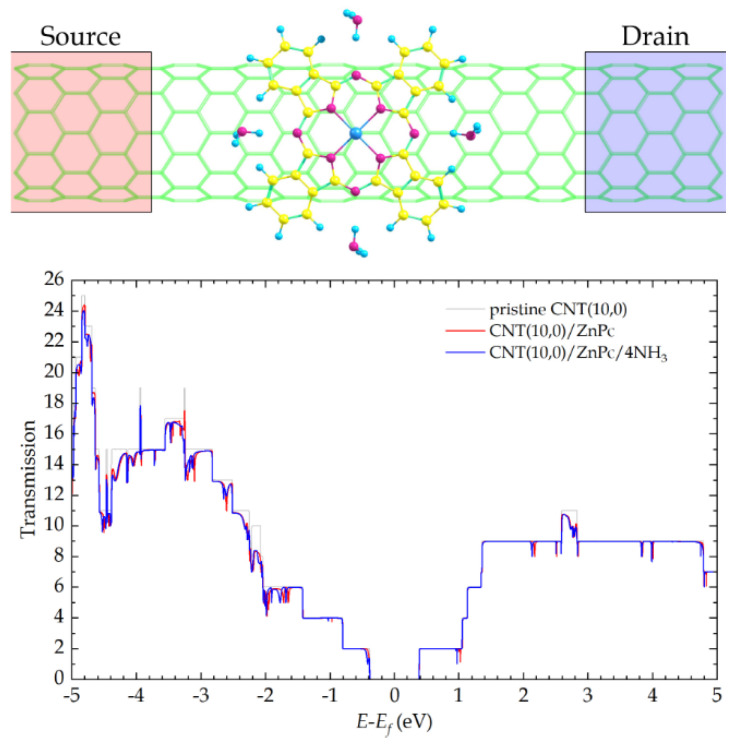
Schematic diagram of the device for calculating the conductivity of hybrids (**top**) and the dependence of the electron transmission coefficient on their energy in the case of the pristine CNT(10,0), CNT(10,0)/ZnPc, and CNT(10,0)/ZnPc/4NH_3_ (**bottom**).

To answer all these questions, we used the NEGF method to calculate the integral conductivity of all the hybrids studied and its change during the adsorption of NH_3_ molecules. For this purpose, the device models were prepared as follows. A fragment of the hybrid structure as one supercell was placed between two electrodes (Source and Drain) (Figure 5). Ideal semi-infinite CNT(10,0) were used as electrodes.

**Figure 6:**
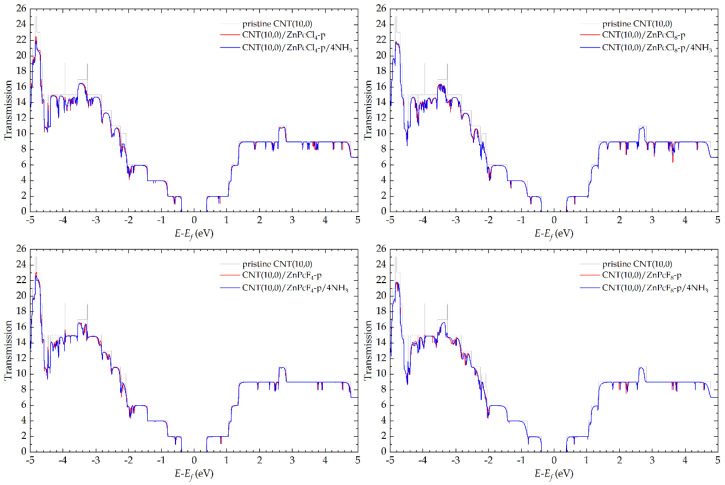
Dependence of the electron transmission coefficient on their energy in the case of CNT(10,0)/ZnPcCl_4_-p, CNT(10,0)/ZnPcCl_8_-p, CNT(10,0)/ZnPcF_4_-p, and CNT(10,0)/ZnPcF_8_-p in the absence and the presence of four adsorbed ammonia molecules.

The procedure for calculating the electrical conductivity consisted of three stages. In the first stage, the parameters of the electronic structure of the electrodes in the form of infinite carbon nanotubes were calculated, including the effective charges of carbon atoms, the Fermi energy *E_f_*, and the Hamiltonian eigenvalues. In the second stage, these parameters were used to calculate the fragment density matrix between the electrodes by integrating Green’s function at zero applied bias. As a result, the dependences of the electron transmission coefficient *T*(*E*) (the probability of electron transition from Source to Drain) on the electron energy *E* were obtained (Figure 5 and Figure 6). In the third stage, the integrated conductivity *G*(*V*) was calculated in a given energy range in the framework of the Landauer formalism [71] as

(3) $$G\left(V\right)=\frac{I}{V}=\frac{2e^{2}}{h}\int_{-eV/2}^{eV}T\left(E\right)dE,$$

where *V* = 10 V is the voltage drop typically used in experiments to measure the sensor response of hybrid compounds based on carbon nanotubes and phthalocyanines.

The addition of zinc phthalocyanines onto the CNT(10,0) surface was accompanied by the appearance of sharp dips in the transmission functions of the pristine carbon nanotube at certain electron energies (Figure 5 and Figure 6). These Fano effects were previously described by Prasongkit et al. [72]. They are associated with the interference of discrete states of macrocycles and continuous bulk states of CNT. Further addition of NH_3_ molecules led mainly to a slight shift of these dips to the left. In a few cases, significant strengthening or weakening of the peaks or even an appearance of new ones was observed (e.g., at −2.6 eV, −3.1 eV, and −3.3 eV in the case of CNT(10,0)/ZnPc or at 3.6 eV in the case of CNT(10,0)/ZnPcCl_8_-p). All these changes are associated with the shift of the electron density from the analyte molecule to the hybrid and should lead to a change in the integral conductivity.

The performed calculations show that in the case of all the hybrids considered, the initial conductivity *G*_0_ decreases to the value *G* upon the adsorption of ammonia molecules (Table 1). This means that in all cases, the sensor response to ammonia is due to a decrease in electrical conductivity (and an increase in resistance). In practice, the sensor response *S* is determined as the ratio of the change in material resistance (*R* − *R*_0_) to the initial resistance *R*_0_. Taking into account that conductivity is the inverse of resistance, we can indicate that

(4) $$S=\frac{R-R_{0}}{R_{0}}=\frac{G_{0}-G}{G}.$$

The calculations have shown that the hybrid with unsubstituted ZnPc had the highest sensor response to ammonia (Table 1). When four halogen atoms were introduced into the macrocycle, the *S* value decreased by about an order of magnitude, and when eight atoms were introduced, it decreased by two orders of magnitude. This correlates with the previously described changes in the band structure of hybrids with halogen substitution in ZnPc. Meanwhile, in the case of hybrids with chloro-substituted macrocycles, the sensor response was slightly higher than that of the hybrids with fluoro-substituted phthalocyanines.

The value of *S* for CNT(10,0)/ZnPc is lower than that observed previously in the experiment (e.g., about 6% at an NH_3_ concentration of 10 ppm) [48]. However, it should be noted that when performing the calculations, we did not consider the functionalization degree of carbon nanotubes with zinc phthalocyanines and, therefore, the adsorbed ammonia concentration. From this point of view, the obtained results are of fundamental importance as they demonstrate how the sensor response of hybrids containing phthalocyanines with different substituents will differ provided that the degree of their functionalization remains the same.

An interesting point is that the binding energy of ammonia molecules to the hybrids does not correlate with the value of the sensor response (Table 1). In the case of CNT(10,0)/ZnPc, CNT(10,0)/ZnPcCl_4_-p and CNT(10,0)/ZnPcF_4_-p, the *E_b_*(NH_3_) values are approximately the same. However, in the case of hybrids with octa-halogen-substituted phthalocyanines, the NH_3_ molecules are bound slightly more strongly. This indicates that the binding energy does not affect the sensitivity of the materials but rather determines their recovery time during which the ammonia molecules desorb. We assume this because the effect of changing the hybrid’s band structure, which occurs when halogen substituents are introduced into the phthalocyanine ring prevails over the effect of shifting the electron density from the ammonia molecule when it binds to the macrocycle.

Another important aspect of the operation of chemical sensors in real conditions is the study of the effect of humidity. This is due to the fact that H_2_O molecules, like NH_3_, are electron donors, and their adsorption on the surface of the CNT-based hybrids also leads to an increase in the initial resistance. Our previous experimental studies of hybrid materials of carbon nanotubes with pyrene-substituted zinc phthalocyanines showed that at relative humidity (RH) of less than 45%, the magnitude of the sensor response of the hybrids to ammonia remained unchanged [40]. However, with a further increase in RH, the sensitivity of the materials became slightly lower. Taking into account that the proposed mechanism of the sensor response to ammonia is the same for hybrids with phthalocyanines, which contain both pyrene and halogen substituents, the same effect of humidity on the sensor response of these hybrids is expected. This means that high RH can distort the ammonia detection results, so it must be monitored to ensure measurement accuracy.

## 4. Conclusions

As a result of the calculations performed, the fundamental influence of the nature of substituents in zinc phthalocyanines in their hybrid materials with carbon nanotubes on the value of the sensor response of these compounds to ammonia was demonstrated. This effect was related to the position of the impurity band formed by the orbitals of the macrocycles relative to the forbidden energy gap of the hybrids. In our previous work [48], we demonstrated using CNT hybrids with phthalocyanines bearing pyrene substituents as examples that the electrons of this impurity band could move to the conduction band due to thermal excitation, increasing the charge concentration in the latter. The interaction of ammonia molecules with hybrids led to a decrease in the impurity band energy and the electrical conductivity of the hybrids, which, in turn, caused their sensor response to ammonia.

In this study, it was shown that the introduction of halogen substituents into zinc phthalocyanine led to a qualitatively different change in the position of the mentioned impurity band compared to unsubstituted ZnPc. This band shifted deep into the valence band, which decreased the conductivity of the hybrids and reduced the effect of the impurity band on the change in their conductivity during the adsorption of ammonia molecules. As a result, the value of the sensor response of the hybrids containing halogen-substituted metal phthalocyanines should be lower compared to compounds with unsubstituted macrocycles and, especially, with macrocycles bearing pyrene substituents.

On the other hand, based on the binding energy values, the introduction of halogen substituents should contribute to an increase in the functionalization degree of carbon nanotubes with phthalocyanines, which, as previously shown, leads to an increase in their sensitivity to gas molecules. However, it should be noted that with the same functionalization degree, the sensor response of carbon nanotubes coated with unsubstituted metal phthalocyanine is about 10 times greater than that of those with tetra-halogen-substituted derivatives. This means that the latter will not produce the same sensor response unless the degree of functionalization is 10 times higher. Shortly, we plan to conduct an experimental study of the degree of functionalization of carbon nanotubes with unsubstituted and halogenated zinc phthalocyanines and the sensor response of the obtained hybrid materials to ammonia to identify agreement with the obtained calculated data.

The obtained results provide a clear understanding of the direction for searching for the optimal molecular structure of metal phthalocyanines in order to enhance the sensor properties of their hybrids with carbon nanotubes. The key concept is that the substituents in the phthalocyanine ring should lead to an increase in the energy of the highest occupied molecular orbital (HOMO) of the macrocycle compared to the unsubstituted macrocycle, which will cause this orbital to fall into the forbidden band of the hybrid as an impurity band. The closer this band is to the conduction band bottom, the higher the sensor response to ammonia will be for the hybrids.

## Figures and Tables

**Table 1:** The binding energies of MC with CNT(10,0) (*E_b_*(MC)) and NH_3_ molecule with CNT(10,0)/MC hybrids (*E_b_*(NH_3_)), band gap (∆*E*_1_), impurity band position (∆*E*_2_), and conductivity (*G*) of hybrids before and after ammonia adsorption, and sensor response of hybrids to ammonia (*S*).

Hybrid	*E_b_*(MC)	*E_b_*(NH_3_)	without NH_3_			with NH_3_			*S*
			Δ*E*_1_	Δ*E*_2_	*G*_0_	Δ*E*_1_	Δ*E*_2_	*G*	
CNT(10,0)/ZnPc	−1.874	−0.186	0.775	0.734	86.62	0.775	0.743	84.19	2.88%
CNT(10,0)/ZnPcCl_4_-p	−2.196	−0.185	0.761	0.934	83.07	0.761	0.944	82.69	0.46%
CNT(10,0)/ZnPcF_4_-p	−1.892	−0.185	0.765	0.923	83.71	0.764	0.923	83.48	0.28%
CNT(10,0)/ZnPcCl_8_-p	−2.526	−0.195	0.762	1.015	81.70	0.763	1.022	81.63	0.08%
CNT(10,0)/ZnPcF_8_-p	−1.925	−0.193	0.769	1.104	82.15	0.768	1.097	82.14	0.02%

Note: energy values are in eV, *G* values are in conductivity quanta (2*e*^2^/*h*).

## Data Availability

Data are contained within the article.

## References

[1] Kong H.X. (2013). Hybrids of carbon nanotubes and graphene/graphene oxide. Curr. Opin. Solid State Mater. Sci..

[2] Wang R., Sun J., Gao L., Xu C., Zhang J. (2011). Fibrous nanocomposites of carbon nanotubes and graphene-oxide with synergetic mechanical and actuative performance. Chem. Commun..

[3] Banimuslem H., Hassan A., Basova T., Esenpınar A.A., Tuncel S., Durmuş M., Gürek A.G., Ahsen V. (2015). Dye-modified carbon nanotubes for the optical detection of amines vapours. Sens. Actuators B Chem..

[4] Bati A.S.R., Hao M., Macdonald T.J., Batmunkh M., Yamauchi Y., Wang L., Shapter J.G. (2021). 1D-2D synergistic MXene-nanotubes hybrids for efficient perovskite solar cells. Small.

[5] Hamdast A., Agbolaghi S., Zeighami M., Beygi-Khosrowshahi Y., Sarvari R. (2019). Butterfly nanostructures via regioregularly grafted multi-walled carbon nanotubes and poly (3-hexylthiophene) to improve photovoltaic characteristics. Polym. Int..

[6] Kar M., Saha S., Sarkar R., Pal S., Sarkar P. (2020). Comparative study on the photovoltaic properties of ZnX (X = S, Se, Te) QD/CNT inorganic/organic hybrid nanocomposites. J. Phys. Chem. C.

[7] Boutahir M., Chenouf J., Mejía-López J., Rahmani A., Chadli H., Rahmani A. (2021). Role of carbon nanotubes as an acceptor to enhance the photovoltaic performances of organic solar cells based on π-conjugated thiophene as a donor materials. Int. J. Energy Res..

[8] Jang Y., Kim S.M., Spinks G.M., Kim S.J. (2020). Carbon nanotube yarn for fiber-shaped electrical sensors, actuators, and energy storage for smart systems. Adv. Mater..

[9] Wang H., Biswas S.K., Zhu S., Lu Y., Yue Y., Han J., Xu X., Wu Q., Xiao H. (2020). Self-healable electro-conductive hydrogels based on core-shell structured nanocellulose/carbon nanotubes hybrids for use as flexible supercapacitors. Nanomaterials.

[10] Mohideen M.M., Liu Y., Ramakrishna S. (2020). Recent progress of carbon dots and carbon nanotubes applied in oxygen reduction reaction of fuel cell for transportation. Appl. Energy.

[11] Cai T., Huang M., Huang Y., Zheng W. (2019). Enhanced performance of microbial fuel cells by electrospinning carbon nanofibers hybrid carbon nanotubes composite anode. Int. J. Hydrogen Energy.

[12] Kim A.R., Vinothkannan M., Song M.H., Lee J.-Y., Lee H.-K., Yoo D.J. (2020). Amine functionalized carbon nanotube (ACNT) filled in sulfonated poly (ether ether ketone) membrane: Effects of ACNT in improving polymer electrolyte fuel cell performance under reduced relative humidity. Compos. Part B Eng..

[13] Liu Y., Gao G., Vecitis C.D. (2020). Prospects of an electroactive carbon nanotube membrane toward environmental applications. Acc. Chem. Res..

[14] Hu P., Lyu J., Fu C., Gong W., Liao J., Lu W., Chen Y., Zhang X. (2019). Multifunctional aramid nanofiber/carbon nanotube hybrid aerogel films. ACS Nano.

[15] Wang Y., Pan C., Chu W., Vipin A.K., Sun L. (2019). Environmental remediation applications of carbon nanotubes and graphene oxide: Adsorption and catalysis. Nanomaterials.

[16] Cheng J., Wang X., Nie T., Yin L., Wang S., Zhao Y., Wu H., Mei H. (2020). A novel electrochemical sensing platform for detection of dopamine based on gold nanobipyramid/multi-walled carbon nanotube hybrids. Anal. Bioanal. Chem..

[17] Qin Y., Sun Y., Li Y., Li C., Wang L., Guo S. (2020). MOF derived Co3O4/N-doped carbon nanotubes hybrids as efficient catalysts for sensitive detection of H2O2 and glucose. Chin. Chem. Lett..

[18] Yao X., Zhang Y., Jin W., Hu Y., Cui Y. (2021). Carbon nanotube field-effect transistor-based chemical and biological sensors. Sensors.

[19] Wang Y., Hu N., Zhou Z., Xu D., Wang Z., Yang Z., Wei H., Kong E.S.-W., Zhang Y. (2011). Single-walled carbon nanotube/cobalt phthalocyanine derivative hybrid material: Preparation, characterization and its gas sensing properties. J. Mater. Chem..

[20] Wang B., Wu Y., Wang X., Chen Z., He C. (2014). Copper phthalocyanine noncovalent functionalized single-walled carbon nanotube with enhanced NH3 sensing performance. Sens. Actuators B Chem..

[21] Şenocak A., Ivanova V., Ganesan A., Klyamer D., Basova T., Makhseed S., Demirbas E., Durmuş M. (2023). Hybrid material based on single walled carbon nanotubes and cobalt phthalocyanine bearing sixteen pyrene moieties as a sensing layer for hydrogen sulfide detection. Dye. Pigment..

[22] Shooshtari M., Salehi A. (2022). An electronic nose based on carbon nanotube-titanium dioxide hybrid nanostructures for detection and discrimination of volatile organic compounds. Sens. Actuators B Chem..

[23] Shooshtari M. (2025). Ammonia gas sensors based on multi-wall carbon nanofiber field effect transistors by using gate modulation. Colloids Surf. A Physicochem. Eng. Asp..

[24] Tanaka K., Cheng G., Nakamura T., Hiraoka K., Tabata H., Kubo O., Komatsu N., Katayama M. (2024). NH3 Gas Sensors Based on Single-Walled Carbon Nanotubes Interlocked with Metal-Tethered Tetragonal Nanobrackets. ACS Appl. Nano Mater..

[25] Satishkumar B.C., Vogl E.M., Govindaraj A., Rao C.N.R. (1996). The decoration of carbon nanotubes by metal nanoparticles. J. Phys. D Appl. Phys..

[26] Muratore C., Reed A.N., Bultman J.E., Ganguli S., Cola B.A., Voevodin A.A. (2013). Nanoparticle decoration of carbon nanotubes by sputtering. Carbon.

[27] Saboor F.H., Ataei A. (2024). Decoration of metal nanoparticles and metal oxide nanoparticles on carbon nanotubes. Adv. J. Chem. Sect. A.

[28] Li L., Li B., Yang G., Li C.Y. (2007). Polymer decoration on carbon nanotubes via physical vapor deposition. Langmuir.

[29] Villemin E., Gravel E., Izard N., Filoramo A., Vivien L., Doris E. (2015). Polymer-Decorated Carbon Nanotubes as Transducers for Label-Free Photonic Biosensors. Chem. Eur. J..

[30] Salem M.A.S., Khan A.M., Manea Y.K., Saleh H.A.M., Ahmad M. (2022). Carbon nanotubes decorated with coordination polymers for fluorescence detection of heavy-metal ions and nitroaromatic chemicals. ACS Omega.

[31] Gotovac S., Yang C.-M., Hattori Y., Takahashi K., Kanoh H., Kaneko K. (2007). Adsorption of polyaromatic hydrocarbons on single wall carbon nanotubes of different functionalities and diameters. J. Colloid Interface Sci..

[32] Zilberman Y., Ionescu R., Feng X., Müllen K., Haick H. (2011). Nanoarray of polycyclic aromatic hydrocarbons and carbon nanotubes for accurate and predictive detection in real-world environmental humidity. ACS Nano.

[33] Basova T.V., Polyakov M.S. (2020). Hybrid materials based on carbon nanotubes and polyaromatic molecules: Methods of functionalization and sensor properties. Macroheterocycles.

[34] Bouanis F.Z., Bensifia M., Florea I., Mahouche-Chergui S., Carbonnier B., Grande D., Léonard C., Yassar A., Pribat D. (2021). Non-covalent functionalization of single walled carbon nanotubes with Fe-/Co-porphyrin and Co-phthalocyanine for field-effect transistor applications. Org. Electron..

[35] Wang L., Pan D., Zhou M., Liang Q., Li Z. (2021). Effect of phthalocyanines supported carbon nanotube for the catalytic oxidation of benzyl alcohol. Solid State Sci..

[36] Li H., Pan Y., Wang Z., Yu Y., Xiong J., Du H., Lai J., Wang L., Feng S. (2022). Coordination engineering of cobalt phthalocyanine by functionalized carbon nanotube for efficient and highly stable carbon dioxide reduction at high current density. Nano Res..

[37] Chen Y., Yao Q., Qu S., Shi W., Li H., Chen L. (2021). Significantly enhanced thermoelectric properties of copper phthalocyanine/single-walled carbon nanotube hybrids by iodine doping. ACS Appl. Mater. Interfaces.

[38] Osama R., Morsy M., Al-Kamel A.N., Mahmoud E.A., Ashery A., El-Sayed A. (2022). Stimulating photodiode characteristics of hybrid ZnPc-MWCNTs. J. Alloys Compd..

[39] Wang Z., Sun Z., Li Q., Zhou M., Liang Q., Li Z., Sun D. (2020). Selective oxidation of styrene to benzaldehyde by cobalt phthalocyanine-multi-walled carbon nanotube composites. Solid State Sci..

[40] Jassim A.H.M., Banimuslem H. (2020). Metal Phthalocyanine Modified Multi Walled Carbon Nanotubes; DC-Conductivity and Optical Properties. Nano Hybrids Compos..

[41] Demir E., Göktug Ö., İnam R., Doyduk D. (2021). Development and characterization of iron (III) phthalocyanine modified carbon nanotube paste electrodes and application for determination of fluometuron herbicide as an electrochemical sensor. J. Electroanal. Chem..

[42] Porto L.S., da Silva D.N., Silva M.C., Pereira A.C. (2019). Electrochemical sensor based on multi-walled carbon nanotubes and cobalt phthalocyanine composite for pyridoxine determination. Electroanalysis.

[43] Ridhi R., Gautam S., Saini G.S.S., Tripathi S.K., Rawat J.S., Jha P. (2020). Amendment in sensing response of Single Walled Carbon nanotube (SWCNT) towards ammonia gas with copper phthalocyanine functionalization. Mater. Today Proc..

[44] Zhang X., Wu Z., Zhang X., Li L., Li Y., Xu H., Li X., Yu X., Zhang Z., Liang Y. (2017). Highly selective and active CO_2_ reduction electrocatalysts based on cobalt phthalocyanine/carbon nanotube hybrid structures. Nat. Commun..

[45] Chen Y., Qu S., Shi W., Yao Q., Chen L. (2020). Enhanced thermoelectric properties of copper phthalocyanine/single-walled carbon nanotubes hybrids. Carbon.

[46] Gai S., Wang B., Wang X., Zhang R., Miao S., Wu Y. (2022). Ultrafast NH3 gas sensor based on phthalocyanine-optimized non-covalent hybrid of carbon nanotubes with pyrrole. Sens. Actuators B Chem..

[47] Ivanova V., Klyamer D., Krasnov P., Kaya E.N., Kulu I., Kostakoğlu S.T., Durmuş M., Basova T. (2023). Hybrid materials based on pyrene-substituted metallo phthalocyanines as sensing layers for ammonia detection: Effect of the number of pyrene substituents. Sens. Actuators B Chem..

[48] Krasnov P., Ivanova V., Klyamer D., Fedorov A., Basova T. (2022). Phthalocyanine-Carbon Nanotube Hybrid Materials: Mechanism of Sensor Response to Ammonia from Quantum-Chemical Point of View. Chemosensors.

[49] Schöllhorn B., Germain J.P., Pauly A., Maleysson C., Blanc J.P. (1998). Influence of peripheral electron-withdrawing substituents on the conductivity of zinc phthalocyanine in the presence of gases. Part 1: Reducing gases. Thin Solid Films.

[50] Brinkmann H., Kelting C., Makarov S., Tsaryova O., Schnurpfeil G., Wöhrle D., Schlettwein D. (2008). Fluorinated phthalocyanines as molecular semiconductor thin films. Phys. Status Solidi Appl. Mater. Sci..

[51] Klyamer D., Sukhikh A., Gromilov S., Krasnov P., Basova T. (2018). Fluorinated metal phthalocyanines: Interplay between fluorination degree, films orientation, and ammonia sensing properties. Sensors.

[52] Kuprikova N.M., Klyamer D.D., Sukhikh A.S., Krasnov P.O., Mrsic I., Basova T.V. (2020). Fluorosubstituted lead phthalocyanines: Crystal structure, spectral and sensing properties. Dye. Pigment..

[53] Bonegardt D., Klyamer D., Sukhikh A., Krasnov P., Popovetskiy P., Basova T. (2021). Fluorination vs. Chlorination: Effect on the Sensor Response of Tetrasubstituted Zinc Phthalocyanine Films to Ammonia. Chemosensors.

[54] Klyamer D., Bonegardt D., Krasnov P., Sukhikh A., Popovetskiy P., Basova T. (2022). Tetrafluorosubstituted Metal Phthalocyanines: Study of the Effect of the Position of Fluorine Substituents on the Chemiresistive Sensor Response to Ammonia. Chemosensors.

[55] Hesse K., Schlettwein D. (1999). Spectroelectrochemical investigations on the reduction of thin films of hexadecafluorophthalocyaninatozinc (F16PcZn). J. Electroanal. Chem..

[56] Elstner M., Porezag D., Jungnickel G., Elsner J., Haugk M., Frauenheim T., Suhai S., Seifert G. (1998). Self-consistent-charge density-functional tight-binding method for simulations of complex materials properties. Phys. Rev. B.

[57] Hourahine B., Aradi B., Blum V., Bonafe F., Buccheri A., Camacho C., Cevallos C., Deshaye M.Y., Dumitrică T., Dominguez A. (2020). DFTB+, a software package for efficient approximate density functional theory based atomistic simulations. J. Chem. Phys..

[58] Lu X., Gaus M., Elstner M., Cui Q. (2015). Parametrization of DFTB3/3OB for magnesium and zinc for chemical and biological applications. J. Phys. Chem. B.

[59] Gaus M., Goez A., Elstner M. (2013). Parametrization and benchmark of DFTB3 for organic molecules. J. Chem. Theory Comput..

[60] Grimme S., Ehrlich S., Goerigk L. (2011). Effect of the damping function in dispersion corrected density functional theory. J. Comput. Chem..

[61] Grimme S., Antony J., Ehrlich S., Krieg H. (2010). A consistent and accurate ab initio parametrization of density functional dispersion correction (DFT-D) for the 94 elements H-Pu. J. Chem. Phys..

[62] Pecchia A., Penazzi G., Salvucci L., Di Carlo A. (2008). Non-equilibrium Green’s functions in density functional tight binding: Method and applications. New J. Phys..

[63] Basiuk V.A., Flores-Sánchez L.J., Meza-Laguna V., Flores-Flores J.O., Bucio-Galindo L., Puente-Lee I., Basiuk E.V. (2018). Noncovalent functionalization of pristine CVD single-walled carbon nanotubes with 3d metal (II) phthalocyanines by adsorption from the gas phase. Appl. Surf. Sci..

[64] Monkhorst H.J., Pack J.D. (1976). Special points for Brillouin-zone integrations. Phys. Rev. B.

[65] Krasnov P.O., Basova T.V., Hassan A. (2018). Interaction of metal phthalocyanines with carbon zigzag and armchair nanotubes with different diameters. Appl. Surf. Sci..

[66] Krasnov P.O., Ivanova V.N., Basova T.V. (2021). Carbon nanotubes functionalized with Zinc (II) phthalocyanines: Effect of the expanded aromatic system and aromatic substituents on the binding energy. Appl. Surf. Sci..

[67] Chia L.S., Du Y.H., Palale S., Lee P.S. (2019). Interaction of Copper Phthalocyanine with Nitrogen Dioxide and Ammonia Investigation Using X-ray Absorption Spectroscopy and Chemiresistive Gas Measurements. ACS Omega.

[68] Amin E.A., Truhlar D.G. (2008). Zn coordination chemistry: Development of benchmark suites for geometries, dipole moments, and bond dissociation energies and their use to test and validate density functionals and molecular orbital theory. J. Chem. Theory Comput..

[69] Alavi D., Mohammadnejad S., Koleini S.M.J. (2022). Mechanism of Zinc Complexation by Alkaline Ligands: A Molecular Modelling Study. J. Min. Environ..

[70] Kaya E.N., Basova T., Polyakov M., Durmuş M., Kadem B., Hassan A. (2015). Hybrid materials of pyrene substituted phthalocyanines with single-walled carbon nanotubes: Structure and sensing properties. RSC Adv..

[71] Datta S. (1995). Electronic Transport in Mesoscopic Systems.

[72] Prasongkit J., Tangsukworakhun S., Jaisutti R., Osotchan T. (2020). Highly sensitive and selective sensing of acetone and hydrogen sulfide using metal phthalocyanine–carbon nanotube hybrids. Appl. Surf. Sci..

